# Patients want their doctors’ help to increase physical activity: a cross sectional study in general practice

**DOI:** 10.1080/02813432.2021.1910670

**Published:** 2021-04-19

**Authors:** Frida Falskog, Ane M. Landsem, Eivind Meland, Bjørn Bjorvatn, Ole P. Hjelle, Thomas Mildestvedt

**Affiliations:** a Faculty of Medicine, University of Bergen, Bergen, Norway; b Department of Global Public Health and Primary Health Care, University of Bergen, Bergen, Norway; c Faculty of Health and Social Sciences, Department of Nursing and Health Sciences, University of South-Eastern Norway, Kongsberg, Norway

**Keywords:** Cross-sectional study, general practice, inactivity, lifestyle change, physical activity, self-rated health

## Abstract

**Background:**

Inactivity is prevalent in patients presenting in general practice, and the health benefits of increased physical activity (PA) are well known. Few studies have explored whether patients want their general practitioner’s (GPs) contribution in facilitating a lifestyle change.

**Objective:**

To identify the characteristics of patients who expect help from their doctor in increasing levels of PA.

**Design:**

We collected data via questionnaires for this cross-sectional study from general practices.

**Setting:**

General practices in Norway, during Spring 2019.

**Subjects:**

A total of 2104 consecutive patients (response rate 75%) participated.

**Main outcome measures:**

The questionnaire included questions about self-rated health, level of physical activity, the desire to become more physically active, and questions about the role of the GP in increasing the level of physical activity in their patients. We analysed our data using Pearson chi-square and binary logistic regression.

**Results:**

Female patients were less active, but their motivation to increase activity and their expectations of receiving help from their doctor were similar to males. Younger patients were more motivated for increased activity, and to manage without help from their doctors. Impaired self-rated health (SRH) was associated with inactivity and, at the same time, with the motivation to become more active with help from general practitioners.

**Conclusion:**

Most patients in the GPs’ office are physically inactive. This study revealed an important message for GPs: in clinical work, emphasise physical activity for health gains, especially for patients with impaired SRH.

**Key Points**
- Four out of five patients attending Norwegian general practice are inactive
- More than 85% of these patients want to increase their physical activity level
- More than 50% would like help from their GP to achieve this goal

## Introduction

The benefits of regular physical activity (PA) on health are well documented [1,2]. Inactivity is estimated to cause more than five million deaths each year and is one of the major reasons for premature deaths in western countries today [3]. Each year, about 15 million people aged 30–70 years die of non-communicable diseases (NCDs) worldwide. Inactivity is among the leading causes of the four main types of NCDs: cardiovascular diseases, cancer, chronic respiratory diseases and diabetes [4]. The World Health Organization (WHO) estimate that physical inactivity is the primary factor in 21-25% of breast and colon cancer cases, in 27% of diabetes and in about 30% of ischaemic heart disease cases [5]. Physical activity has also been associated with improvements in chronic pain [6] and musculoskeletal disorders, such as knee osteoarthritis [7].

According to Norwegian reports, two-thirds or more of the general population do not meet the Norwegian Directorate of Health’s recommendations for PA, which are 150 min of activity at moderate to vigorous intensity per week, including muscle strengthening activities on two or more days [8–10]. However, three quarters of the general population say they are aware of the national recommendations [10]. National and international recommendations for PA are made to provide scientifically supported measures for the policymakers and stakeholders when guiding the population towards increased levels of PA. For some individuals; however, the way these recommendations are conveyed can appear almost impossible to achieve, thus creating an unnecessary barrier to PA [11].

In Norway, all citizens are listed with a general practitioner (GP). In 2018, about 4800 GPs were registered and they had, on average, 1100 patients each on their patient list [12]. Seven out of 10 Norwegians consult their GP within a year. On average, a Norwegian GP has 2.7 consultations per citizen per year [13]. The system promotes doctor-patient continuity and GPs act as gatekeepers to the secondary health care system.

Six out of 10 Norwegians consider their GP and other health professionals as reliable sources of lifestyle advice [10]. A systematic review reported that promoting PA at the GP’s office significantly increased self-reported levels of PA after 12 months [14]. Studies from Sweden and New Zealand reported increases in, and some adherence to PA when exercise was prescribed by the GP [15,16]. Verbal advice, like brief interventions, are less expensive than standard care [17]. Furthermore, positive outcomes are associated with autonomy-supportive interventions [18]. General practitioners are therefore in a unique position to promote patients’ internal reasons for change, to build motivation on former mastery experiences and to foster long-term lifestyle changes [19].

Three of the authors of the present study (TM, BB and EM) revealed in a former study that patients in primary care exhibited impaired self-rated health (SRH) compared with the general population [20]. Improved SRH is associated with increased PA both in cross-sectional and in longitudinal studies [21]. Self-rated health is a consistent predictor of mortality and morbidity [22,23]. Encouraging PA, therefore, may be an important health promoting task for GPs.

The main objective of this study was to identify the prevalence and characteristics of patients at the GP’s office who: (1) are classified as inactive; (2) want to increase their PA and, (3) want help from their GP to make a change in their activity level. We also wanted to discern how SRH was associated with the inclination to become more active and to want help from the GP in doing so.

## Material and methods

### Selection of study subjects

Our study was based on self-reported data in questionnaires collected from patients in GP waiting rooms from February through May 2019. The questionnaires were distributed by medical students in their final year, who were deployed at different general practices in Western Norway. Each student was asked to collect 30 questionnaires from patients aged 18 years and older, in the waiting room. In practice, this was often done by the secretaries who asked 30 consecutive patients if they wanted to participate, regardless of which doctor they were going to. The completed questionnaires were submitted in a closed envelope with no identifiable marks. Table 1 presents the participants according to gender and age group.

**Table 1.** Characteristics of 2104 adult patients visiting their GP, in western Norway, 2019.

	Total *n*	%	(*n*)
Gender	*n* = 2099		
Female		61.3	(1286)
Male		38.7	(813)
Age	*n* = 2104		
18–32 years		21.2	(447)
33–47 years		27.8	(584)
48–62 years		24.5	(515)
63–100 years		26.5	(558)
Self-rated health	*n* = 2033		
Poor (poor and fairly poor)		24.9	(506)
Good		34.8	(707)
Excellent (very good and excellent)		40.3	(820)
Imagine an average week: how often are you physically active for at least 30 min a day, so you get sweaty / breathless?	*n* = 2058		
< 5 times/week		80.6	(1659)
≥ 5 times/week		19.4	(399)
Would you like to become more physically active?	*n* = 2035		
Yes		80.2	(1633)
No		19.8	(402)
I want help from the GP to increase my physical activity level	*n* = 2015		
Disagree		55.9	(1126)
Agree		44.1	(889)
I want the GP to focus more on physical activity	*n* = 2015		
Disagree		38.7	(780)
Agree		61.3	(1235)
I would choose increased physical activity over medicine if possible	*n* = 2029		
Disagree		6.3	(128)
Agree		93.7	(1901)

### Measurement

The patients answered a one-page questionnaire, in Norwegian, that consisted of questions on PA and their expectations of their GP regarding PA. We did not ask about their reasons for visiting the GP, and patients were not compensated for their participation.

To compare our findings with those from previous Norwegian surveys, we used questions and definitions from a large study about physically inactive adults in Norway published in 2009 [10]. For comparison reasons our PA questions were in line with the national recommendations for PA present in 2009, and not the more recent 2014 recommendations. As Table 1 shows, we applied two different questions to assess physical inactivity. The first question was: ‘Imagine an average week: How often are you physically active for at least 30 min per day so that you perspire and are out of breath?’. There were five response alternatives: 0 times per week; 1–2 times per week; 3–4 times per week; 5–6 times per week, and 7 or more times per week. The participants were classified as inactive if their response was <5 times per week, and thereby did not fulfil, or nearly fulfil, the national recommendations for PA in place in Norway in 2009 [24].

Inactive patients were further defined as either potentially active or not potentially active using the question: ‘Would you like to become more physically active?’ (yes/no). This question was also used in a Norwegian survey from 2009 [10]. In addition, we asked for the patients’ SRH on a 5-point Likert scale from poor to excellent. We rescaled the SRH predictor into three categories in order to gain statistical power in our analyses. The ‘poor’ category had rather few valid answers and the confidence intervals (CIs) of some of the comparisons would become wide and difficult to interpret without doing so. Rescaling SRH into three categories is also common in the SRH-literature. We dichotomized two of the outcomes in order to achieve methodological unity (logistic regression). The Likert scales were without a neutral middle value, allowing us to dichotomize and preserving honesty to the data. We created four age groups: 18–32 years; 33–47 years; 48–62 years, and 63–100 years.

The extent to which patients wanted help from their GP was assessed by the statement: ‘I want help from the GP to increase my PA’. We used this statement to further define our group into the category ‘potentially active who want help from their GP’. A second statement: ‘I want the GP to focus more on PA’ was also added to assess patients’ attitudes towards their GP focusing on lifestyle changes. Furthermore, we explored whether the patients would prefer PA rather than medication with the statement: ‘I would choose increased PA over medication if possible’. All three of these statements had responses on a 6-point Likert scale, which identified how much patients agreed or disagreed with each claim. We dichotomised the variables with the three agree alternatives as one group and the three disagree alternatives as the other group.

### Statistics

We used IBM SPSS Statistics version 25 to perform the data analyses. Differences in the characteristics of patients were investigated using cross table Pearson chi-square and binary logistic regression analyses. Significance level was set to 0.05.

## Results

Of 83 medical students deployed, 70 submitted data for the study. On average, each student collected data from 32 patients (range 7–40). The response rate of 75% was calculated from reports from 64 students. The six remaining students did not systematically report the number of patients who declined to participate.

The total number of participants was 2104, excluding patients under the age of 18 and those who did not report their age. The mean age of participants was 49.0 years (SD 17.5) (range 18–92) and 61.3% of the participants were women.

Four out of five patients in the study population were defined as physically inactive, by either not fulfilling or nearly fulfilling the Norwegian recommendations for PA from 2009 (Table 1). Of these, 85.7% wanted to increase their PA, which we defined as potentially active, and 50.5% of this group wanted their GPs to help them to increase their activity level. Further, in the total study population, 44.1% wanted help from their GP to increase their level of PA; 61.3% wanted their GP to focus more on PA and 93.7% preferred PA over medication if possible (Table 1).

Table 2 presents the six outcomes: inactive, potentially active, potentially active who want help from the GP to increase their level of PA, patients who responded to the statements ‘I want help from the GP to increase my physical activity’, ‘I want the GP to focus more on physical activity’, ‘I would choose increased physical activity over medication if possible’. The table shows the association between these outcomes and the predicting variables of gender, age and SRH. With the exception of the statement: ‘I would choose increased physical activity over medication if possible’, all outcomes were significantly associated with gender.

**Table 2.** Physical activity group and attitude towards change among the patients visiting their GP according to their gender, age and SRH.

	Inactive^a^	Potentially active^b^	Potentially active wanting help^c^	I want help from the GP to increase my physical activity level	I want the GP to focus more on physical activity	I would choose increased physical activity over medication if possible
Gender	*p* < 0.01	*p* = 0.04	*p* < 0.01	*p* < 0.01	*p* < 0.01	*p* = 0.53
% of female (*n*)	83.2 (1044)	87.1 (883)	45.2 (387)	40.1 (490)	58.9 (721)	93.5 (1157)
% of male (n)	76.5 (611)	83.4 (501)	59.9 (297)	50.4 (397)	64.9 (510)	94.2 (741)
Age	*p* < 0.01	*p* < 0.01	*p* < 0.01	*p* < 0.01	*p* = 0.29	*p* = 0.16
% of 18–32 years (*n*)	76.4 (339)	92.0 (310)	41.2 (127)	36.5 (162)	58.2 (258)	94.8 (419)
% of 33–47 years (*n*)	85.1 (492)	92.0 (447)	46.0 (202)	40.9 (234)	63.2 (361)	93.5 (534)
% of 48–62 years (*n*)	78.5 (399)	86.2 (338)	53.9 (178)	45.7 (229)	59.9 (299)	94.9 (479)
% of 63–100 years (*n*)	81.3 (429)	72.5 (292)	63.8 (178)	53.0 (264)	63.1 (317)	91.8 (469)
Self-rated health	*p* < 0.01	*p* = 0.11	*p* < 0.01	*p* < 0.01	*p* = 0.27	*p* < 0.01
% of poor (*n*)	83.1 (413)	87.0 (348)	63.8 (215)	57.1 (273)	62.8 (302)	90.5 (436)
% of good (*n*)	86.4 (597)	87.3 (510)	53.0 (267)	47.5 (324)	62.4 (424)	93.4 (641)
% of excellent (*n*)	74.5 (602)	83.4 (492)	37.6 (180)	32.7 (260)	58.9 (467)	96.1 (767)

Analysed with cross table analyses and Chi^2^ significance tests.

^a^ Patients visiting their GP who are physically inactive; less than 5 times a week with at least 30 min a day so you perspire and are out of breath.

^b^ Patients visiting their GP who are physically inactive; less than 5 times a week with at least 30 min a day so you perspire and are out of breath, who want to increase their level of physical activity.

^c^ Patients visiting their GP who are physically inactive; less than 5 times a week with at least 30 min a day so you perspire and are out of breath, who want to increase their level of physical activity and want help from the GP to achieve this goal.

Women were more likely to be physically inactive and, of these, a higher proportion wanted to increase their activity levels, compared to men. Those who wanted to increase their activity level were defined as potentially active. On the other hand, male gender was positively associated with the other outcomes (Table 2).

Further, we saw statistically significant differences due to age across all outcomes, except in the statements: ‘I want the GP to focus more on physical activity’, and ‘I would choose increased physical activity over medication if possible’. Younger age was associated with the outcome ‘Potentially active’, and older age with the outcomes ‘Potentially active wanting help from the GP’ and ‘I want help from the GP to increase my physical activity level’. Finally, we saw statistically significant differences due to SRH across all outcomes except for inactive participants who intended to increase their level of PA, and the statement: ‘I want the GP to focus more on physical activity’.

In logistic regression analyses, we found associations for the outcome inactivity and the predicting variables of gender and SRH. In the full model analysis, in which we adjusted for the inter-relation between gender, age, and SRH, we confirmed that women were less active than men, and that lower levels of SRH were associated with inactivity. Age was not a significant predictor in the full model analysis (Table 3).

**Table 3.** Unadjusted and full model binary logistic regression analysis for the relationship between inactivity^a^ and the predicting variables.

	Unadjusted		Full model^b^	
	OR	95% CI	OR	95% CI
Gender				
Female	1.52	1.22;1.90	1.58	1.25;1.99
Male	1		1	
Age				
18–32 years	0.75	0.55;1.02	0.78	0.56;1.08
33–47 years	1.32	0.96;1.81	1.33	0.95;1.85
48–62 years	0.85	0.62;1.15	0.83	0.61;1.15
63–100 years	1		1	
Self-rated health				
Poor	1.68	1.27;2.23	1.68	1.25;2.24
Good	2.17	1.66;2.84	2.20	1.67;2.89
Excellent	1		1	

^a^ Patients visiting their GP who are physically inactive; less than 5 times a week with at least 30 min a day so you perspire and are out of breath.

^b^ Adjusting for the inter-relation between gender, age, and SRH.

We found more potentially active participants among young patients and revealed that those with impaired SRH intended to be more active compared to patients with excellent SRH. Gender was not a significant predictor in the full model analysis (Table 4).

**Table 4.** Unadjusted and full model binary logistic regression analysis for the relationship between potentially active^a^ and the predicting variables.

	Unadjusted		Full model^b^	
	OR	95% CI	OR	95% CI
Gender				
Female	1.35	1.01;1.78	1.06	0.78;1.43
Male	1		1	
Age				
18–32 years	4.37	2.78;6.84	5.20	3.23;8.37
33–47 years	4.36	2.94;6.46	4.87	3.23;7.34
48–62 years	2.38	1.66;3.41	2.52	1.74;3.66
63–100 years	1		1	
Self-rated health				
Poor	1.33	0.93;1.92	1.80	1.23;2.64
Good	1.37	0.99;1.90	1.75	1.24;2.47
Excellent	1		1	

^a^ Patients visiting their GP who are physically inactive; less than 5 times a week with at least 30 min a day so you perspire and are out of breath, who want to increase their level of physical activity.

^b^ Adjusting for the inter-relation between gender, age, and SRH.

Participants intending to increase PA and wanting help from their GP were more likely to be male, older and with impaired SRH. These findings were also sustained in the full model analysis (Table 5).

**Table 5.** Unadjusted and full model binary logistic regression analysis for the relationship between potentially active who want help from the GP to increase their level of PA^a^ and the predicting variables.

	Unadjusted		Full model^b^	
	OR	95% CI	OR	95% CI
Gender				
Female	0.55	0.44;0.69	0.59	0.47;0.75
Male	1		1	
Age				
18–32 years	0.40	0.29;0.56	0.57	0.40;0.82
33–47 years	0.48	0.36;0.66	0.61	0.44;0.84
48–62 years	0.66	0.48;0.92	0.75	0.54;1.06
63–100 years	1		1	
Self-rated health				
Poor	2.93	2.19;3.91	2.68	1.99;3.61
Good	1.87	1.45;2.41	1.73	1.33;2.25
Excellent	1		1	

^a^ Patients visiting their GP who are physically inactive; less than 5 times a week with at least 30 min a day so you perspire and are out of breath, who want to increase their level of physical activity, and want help from the GP to achieve this goal.

^b^ Adjusting for the inter-relation between gender, age, and SRH.

## Discussion

The present study revealed that four out of five patients visiting general practice were physically inactive. The majority of these intended to become more active, and half of them wanted help from their GP to achieve this goal. Female patients were more inactive than male patients, but their motivation to increase PA and their desire for the GPs’ help were similar to those of male patients. Younger patients intended to be more active, and to manage without help from their doctors. Impaired SRH was associated with inactivity and, at the same time, with the motivation to be more active with help from GPs.

### Comparison with existing studies

Our finding that 80% of Norwegian patients visiting their GP are inactive is not in line with previous studies which report decreases in levels of inactivity in the general population [9]. Norwegian national mappings report a fall in levels of inactivity from 80% in 2009 to 68% in 2015 for people who do not meet national PA recommendations [9,27]. These mappings used both subjective and objective forms of measurement and classified inactive as not meeting the national guidelines in force at the time (the 2009 guidelines changed in 2014). Their objective measurement was an accelerometer worn by the participants which measured both frequency, intensity and duration of activity during the week [9,27]. We used only subjective measurements and our definition of inactivity was based on the guidelines from 2009 [24].

Our contradictory findings can be explained by the fact that our study population consisted of patients visiting their GP, who reported lower SRH than the general population [20]. Lower SRH has been associated with impaired levels of PA [21]. Our findings might further be explained by us not including the new guidelines for PA issued in 2014, where the option of high-intensity training for 75 min a week was added. In addition, the conflicting findings may be explained by the lack of detail regarding the quality of PA in our questionnaire.

Women in our study were more likely than men to be inactive, which is in contrast with previous studies which found that women were more likely to meet the national recommendations for PA [9,27]. In the course of a year, a greater proportion of women in Norway visit their GP [13], often during life events associated with lower activity levels, such as pregnancy and menopause [28], which may explain the present finding.

The prevalence of potentially active participants in our study was larger than in previous population reports. A report on the general population from 2009 showed that 76% of respondents were potentially active, while we revealed 86% to be so [10]. In line with findings from that 2009 report, people in the oldest age group were less likely to be potentially active. The prevalence of illness, chronic diseases and the fear of accidents increase with age, and this might contribute to older people feeling less inclined to increase their level of PA [29]. Interestingly, the participants in our study who were potentially active and who wanted help from their GP were in the older age group. This implies that when older people want to increase their activity level, they want their GPs’ help.

As this study revealed, many patients intended to increase their activity level. We believe our findings indicate that the clinical population would be broadly positive if GPs addressed PA as a subject in consultations on a larger scale than they do today, although not all patients would want to commit to the idea of receiving help. However, we should be aware that supporting patient’s autonomous motivation is associated with a greater adherence to lifestyle changes [18].

In line with previous studies, we saw that high levels of SRH were associated with high levels of activity [21]. We identified, however, that inactive patients were most likely to belong in the middle group: those with good SRH. This implies that there are large numbers of inactive people in the poorer levels of SRH, not just in the poorest. Further, potentially active patients reported impaired SRH, as did participants with higher expectations of help from their GP. Previous studies demonstrated SRH to be a valid measurement of health in the population [30,31], and other studies have shown the positive implication of PA for SRH [32]. Thus, it is important for GPs to focus on patients with impaired SRH when encouraging increased PA.

### Strengths and limitations

The strengths of our study were the high response rate and the large study population. Our only exclusion criterion was age <18, and therefore, we believe that the study population is representative of the Norwegian adult population visiting their GP. We must, however, consider selection bias as the questionnaire was only produced in Norwegian, thus excluding non-Norwegian speakers. Our questionnaire included well-established scales and questions that have previously proved to be valid and reliable [10], and they were answered consistently overall.

A limitation in our study, in common with other studies exploring self-evaluated PA assessments, was that participants may have different conceptions of ‘physical activity’ and ‘exercise’ [25]. Another challenge is national differences in recommendations for PA, exercise and other health promoting measures, and how these develop with new findings from PA research [26]. This makes it difficult to compare new results with previous findings [25].

We used the national recommendations from 2009 to define inactivity, even though the recommendations from 2014 also included 75-min intensive exercise. We may have overestimated the inactive group by including people who did high-intensity exercise 2–4 times weekly. However, our decision was a deliberate one as our goal was to be able to compare our results with existing studies. If we had intended to measure inactivity based on the new guidelines, a much higher precision of our questionnaire regarding frequency, intensity and duration of PA would have been demanded.

We acknowledge that confounding by factors not included in this study is a threat to the internal validity of the study. In particular, education level and other factors related to socioeconomic status are associated both with SRH and physical activity measures.

This study was conducted in Norwegian primary health care practices, where GPs are generally the first-line service. The structure of health care systems varies between countries, but we believe our results can be generalised to countries with a similar primary care structure as Norway.

### Implication for research and practice

This study revealed that GPs meet many patients every day who intend to increase their level of PA. GPs, therefore, are in an ideal position to guide their patients, and to help foster intrinsic motivation to facilitate change [19]. As nearly the whole clinical population visiting GPs in our study would prefer PA rather than medication, GPs should bring this alternative up more often. Prescribed exercise has been shown to increase levels of activity both in the short and the long term [15,16].

While guiding patients it is important for GPs to clearly convey that any increase in PA provides health benefits, without placing too much focus on national recommendations which could create a barrier for some inactive patients [11]. GPs should focus on patients with impaired SRH as they are a group with large health gain potential. They are also heavily represented in the group of potentially active patients who want help from their GPs to increase their level of PA. Further research is required to explore effective interventions for these patients and how they can be integrated into busy GP practices.

## Conclusion

Most patients in the GPs office are physically inactive. The GP should emphasize PA in clinical consultations, especially when faced with patients who have impaired self-rated health.

The focus is expected by the patients, and has the potential to improve the physical and mental health of the individual patient, reduce morbidity and mortality in the population and help decrease the social costs of inactivity.
